# Disparities in Patterns of Health Care Travel Among Inpatients Diagnosed With Congestive Heart Failure, Florida, 2011

**DOI:** 10.5888/pcd12.150079

**Published:** 2015-09-17

**Authors:** Peng Jia, Imam M. Xierali

**Affiliations:** Author Affiliation: Imam M. Xierali, Louisiana State University, Baton Rouge, Louisiana, and Association of American Medical Colleges, Washington, DC.

## Abstract

**Introduction:**

Congestive heart failure (CHF) is a major public health problem in the United States and is a leading cause of hospitalization in the elderly population. Understanding the health care travel patterns of CHF patients and their underlying cause is important to balance the supply and demand for local hospital resources. This article explores the nonclinical factors that prompt CHF patients to seek distant instead of local hospitalization.

**Methods:**

Local hospitalization was defined as inpatients staying within hospital service areas, and distant hospitalization was defined as inpatients traveling outside hospital service areas, based on individual hospital discharge data in 2011 generated by a Dartmouth–Swiss hybrid approach. Multiple logistic and linear regression models were used to compare the travel patterns of different groups of inpatients in Florida.

**Results:**

Black patients, no-charge patients, patients living in large metropolitan areas, and patients with a low socioeconomic status were more likely to seek local hospitalization than were white patients, those who were privately insured, those who lived in rural areas, and those with a high socioeconomic status, respectively.

**Conclusion:**

Findings indicate that different populations diagnosed with CHF had different travel patterns for hospitalization. Changes or disruptions in local hospital supply could differentially affect different groups in a population. Policy makers could target efforts to CHF patients who are less likely to travel to seek treatment.

## Introduction

Congestive heart failure (CHF) is a major public health problem in the United States. In 2010, it was the leading cause of hospitalization in the population older than 85 years and the second leading cause of hospitalization for people aged 65 to 84 years (1). To provide all CHF patients high-quality and timely services, hospital resources must be made equally accessible for the entire population (2). However, regardless of accessibility, patients do not always go to their nearest hospitals; this fact confounds the local supply–demand relationships. Therefore, it is critical to understand the travel patterns of CHF patients and the factors that influence those patterns.

Research on the travel patterns of hospital patients has increased during the last 2 decades. Long travel distance and travel time to hospitals and other medical facilities, in addition to depriving local hospitals of revenue and deteriorating the patient–doctor relationship (3–5), are associated with decreased health-related quality of life and increased mortality risk, especially with regard to the use of emergency services (6,7). For example, long travel time to a dialysis unit is negatively associated with patients’ health-related quality of life (6). Likewise, increased travel time to the nearest hospitals providing surgery, chemotherapy, or radiotherapy for cancer patients made patients less likely to receive those services (8). A review article of determinants of delivery-service use found that distance to health services was both a disincentive to seeking care and an obstacle to receiving care (9). Compared with its role in developed countries, travel distance affects the use of health care services more in low- and middle-income countries, especially in rural areas (10).

Many factors affect patients’ decisions to seek medical services within or outside of their residence counties. In one study, patients aged 75 or older were less likely to travel across county boundaries to seek treatment (4). Racial/ethnic disparities in travel patterns for care seeking were reported by several studies, and it was consistently found that, compared with whites, nonwhites were less likely to travel long distances for hospitalization for referral-sensitive surgeries or ambulatory care-sensitive admissions, even when they were severely ill (11–13). Lack of health insurance is a crucial determinant of patients’ choosing to stay in their residence county for hospitalization (13). In addition to age, race/ethnicity, and health insurance status (13–16), other factors that influence travel patterns are sex, severity of illness (13), patient location (15), and socioeconomic status (SES) (11,17). 

Traveling to hospitals outside a patient’s county of residence is often classified as a longer-than-normal trip (18). However, some patients live closer to hospitals outside than inside their county of residence (3). Therefore, administrative unit boundaries may not represent underlying local patterns of hospital travel as well as do hospital service areas (HSAs) (19), a series of functional areas in which most patients live and go to a hospital or group of hospitals (20). In this study, being hospitalized in a patient’s HSA is defined as local hospitalization, and traveling outside a patients’ HSA is defined as distant hospitalization.

Distinct from most empirical studies, which have focused on minority groups that had less accessibility to hospitals and other medical resources, the objective of this study was to explore the nonclinical characteristics of CHF patients attributable to distant instead of local hospitalization. We integrated all factors that influence the travel patterns of CHF patients and examined them to determine whether disparities in patterns exist; in addition to demographic characteristics, we considered the presence of comorbidities. Conclusions will aid in identifying the factors associated with patients’ travel patterns and informing policy makers of populations that are most affected by the changes in local hospital resources, such as reduction in the number of beds or hospital closure.

## Methods

Data from the Agency for Healthcare Research and Quality’s (AHRQ’s) State Inpatient Database (SID), part of the Healthcare Cost and Utilization Project (HCUP), which includes individual discharge records from all hospitals in Florida during the year 2011, was used in this study (21). Florida is considered an ideal region for examining the travel patterns of inpatients (3). Each record includes a range of demographic and socioeconomic characteristics. Using the zip code of residence of inpatients, individual discharge records can be geocoded at the zip code level. “Patient” and “inpatient” are interchangeably used in this study.

Clinical Classification Software developed by AHRQ was used to identify all hospital discharges diagnosed with CHF (coded as 108, nonhypertensive), which ranked second by the number of discharges among all diagnosis groups in Florida in 2011. A total of 62,451 discharge records from 221 general hospitals were used in this study. The primary and secondary road network with speed limit information in Florida was obtained from the Florida Department of Transportation, manually corrected in ArcGIS version 10.2 (ESRI), and used for calculating the travel time from the population-weighted centroid of postal zone to hospital (22).

Florida was divided into 78 HSAs on the basis of all individual discharges from 221 hospitals in Florida during 2011, using a Dartmouth–Swiss hybrid method (3). A discharge from one HSA to the same HSA was defined as local hospitalization, and a discharge from one HSA to a different HSA was defined as distant hospitalization. Multiple logistic regression models were used to estimate the association of local hospitalization with race/ethnicity, with local/distant hospitalization assigned as the dependent variable (1 for local hospitalization and 0 for distant hospitalization). Six exclusive racial/ethnic categories were used: white, black, Hispanic, Asian, Native American, and other.

Potential confounding factors were adjusted to remove their effects on the disparities among races/ethnicities, including age, sex, payer, patient location (large metropolitan area [≥1 million residents], small metropolitan area [<1 million residents], micropolitan area, or rural area), SES (measured by the median household income for patient’s zip code), severity of illness (indirectly determined by surgery received or not received and death as an outcome), length of stay, and presence of comorbidities. Race/ethnicity was controlled for while examining the associations of local hospitalization with other factors. All categories of each factor were subsequently used as reference categories, to which the odds ratios (ORs) of all the other categories were compared.

In addition to the dichotomous proxy for local or distant hospitalization, candidate measures of time from the population-weighted centroid of each postal zone to each hospital were actual and excess travel time. On the basis of the primary and secondary road network with speed limits, actual travel time for a given discharge record was calculated along the shortest driving path from the population-weighted centroid of the postal zone to which that record was discharged to the hospital from which that record was discharged. Second, excess travel time for a given discharge record was computed by subtracting time spent from population-weighted centroid of that postal zone to the nearest hospital from the actual travel time.

Two measures alternated as a continuous dependent variable in linear regression models, which were used to study the association of travel patterns with the same set of factors included in multiple logistic regression models. All values in 4 primary categories (race/ethnicity, payer, patient location, and SES) were converted and used as “dummy” variables in linear regression models. Results from both multiple logistic and linear regression models were all tested at the 90%, 95%, and 99% confidence levels.

## Results

Of all CHF discharges, 18.3% were black, and 14.4% were Hispanic; 0.49% were Asian, and 0.1% were Native American (Table 1). Approximately 78.8% (49,234) of the CHF hospitalizations occurred in the inpatients’ local HSAs during 2011.

**Table 1:** Descriptive Statistics, Odds of Local Hospitalization, and Predictors of Travel Time for Patients Diagnosed With Congestive Heart Failure (N = 62,451), Florida, United States, 2011

Predictor	Total Discharge, n	Crude Rate, %	OR (95% CI)	Standard Coefficient, Travel Time	Standard Coefficient, Excess Time
**Sex**					
Male	32,634	77.6	1.00	1	1
Female	29,817	80.2	1.10 (1.06–1.14)	−0.017	−0.018
**Age**	—	—	1.014 (1.012–1.016)	−0.096	−0.090
**Length of stay**	—	—	0.99 (0.987–0.994)	—	—
**Race/ethnicity**					
White	41,008	78.5	1.00	1	1
Black	11,402	79.9	1.24 (1.17–1.32)	−0.031	−0.019
Hispanic	9,010	79.2	1.03 (0.97–1.10)	−0.027	0.001
Asian	305	81.0	1.31 (0.98–1.76)	−0.009	−0.007
Native American	75	81.3	1.29 (0.72–2.31)	0.003	0.004
Other	651	77.1	0.99 (0.82–1.20)	0.006	0.008
**Payer**					
Medicare	49,799	79.7	1.00	1	1
Medicaid	4,865	78.0	1.15 (1.06–1.25)	−0.013	−0.011
Private	4,074	70.0	0.77 (0.71–0.83)	0.042	0.046
Self-pay	1,735	78.5	1.24 (1.09–1.40)	−0.004	−0.003
No charge	681	79.7	1.27 (1.04–1.54)	−0.016	−0.013
Other	1,297	76.2	0.92 (0.80–1.05)	0.007	0.006
**Location/area^a^**					
Large metropolitan	37,446	79.7	1.00	1	1
Small metropolitan	20,035	78.7	0.94 (0.90–0.99)	0.076	0.015
Micropolitan	3,324	76.4	0.81 (0.74–0.88)	0.141	0.084
Rural	1,646	66.7	0.50 (0.45–0.56)	0.224	0.184
**Income quartile**					
1st	23,563	79.0	1.00	1	1
2nd	19,156	79.3	0.99 (0.95–1.04)	0.043	0.019
3rd	15,232	78.3	0.92 (0.87–0.97)	0.078	0.047
4th	4,500	77.9	0.90 (0.83–0.98)	0.064	0.039
**Severity**					
Not surgery	56,208	79.8	1.00	1	1
Surgery	6,243	70.5	0.66 (0.62–0.71)	0.057	0.059
Not died	60,764	78.8	1.00	1	1
Died	1,687	79.5	0.92 (0.81–1.04)	−0.006	−0.004
Not AIDS	62,202	78.8	1.00	—	—
AIDS	249	83.9	1.66 (1.18–2.34)	—	—
Not chronic pulmonary disease	36,903	78.0	1.00	1	1
Chronic pulmonary disease	25,548	80.0	1.14 (1.09–1.18)	−0.030	−0.030
Not hypertension	13,448	77.5	1.00	1	1
Hypertension	49,003	79.2	1.04 (0.99–1.09)	−0.011	−0.015
Not liver disease	60,750	78.9	1.00	—	—
Liver disease	1,701	75.3	0.91 (0.81–1.02)	—	—
Not neurological disorders	58,030	78.7	1.00	1	1
Neurological disorders	4,421	80.8	1.09 (1.01–1.18)	−0.010	−0.008
Not obesity	51,069	78.9	1.00	1	1
Obesity	11,382	78.4	1.08 (1.03–1.14)	−0.010	−0.013
Not weight loss	60,381	79.0	1.00	1	1
Weight loss	2,070	74.0	0.79 (0.72–0.88)	0.008	0.010
Not solid tumor without metastasis	61,446	78.8	1.00	1	1
Solid tumor without metastasis	1,005	81.1	1.15 (0.98–1.35)	−0.010	−0.008
Not coagulopathy	58,429	78.9	1.00	1	1
Coagulopathy	4,022	77.5	1.13 (1.02–1.26)	0.010	0.011
Not deficiency anemias	40,734	78.7	—	1	1
Deficiency anemias	21,717	79.1	—	−0.008	−0.008
Not diabetes with chronic complications	56,549	78.9	—	1	1
Diabetes with chronic complications	5,902	77.8	—	−0.014	−0.014
Not hypothyroidism	51,447	78.7	—	1	1
Hypothyroidism	11,004	79.3	—	0.010	0.010
Not fluid and electrolyte disorders	43,706	79.1	—	1	1
Fluid and electrolyte disorders	18,745	78.1	—	0.016	0.016
Not psychoses	60,666	78.9	—	1	—
Psychoses	1,785	77.8	—	−0.009	—
Not renal failure	37,176	78.9	—	1	1
Renal failure	25,275	78.8	—	0.015	0.012
Not valvular disease	62,164	78.9	—	1	1
Valvular disease	287	74.9	—	−0.010	−0.012

Abbreviations: — , not applicable; AIDS, acquired immune deficiency syndrome; CI, confidence interval; OR, odds ratio.

a A large metropolitan area was 1 million or more residents, and a small metropolitan area was fewer than 1 million residents.

When logistic models controlled for all known predictors for travel patterns (Table 1), female inpatients were more likely to stay within their HSAs for hospitalization than were male patients. Linear models consistently indicated that female patients spent 1.7% less travel time and 1.8% less excess travel time than male patients. Odds of local hospitalization increased with age, and local patients tended to have a shorter stay in hospital. Distant inpatients were more likely to receive surgery than their local counterparts. However, no significant differences in death rate at the time of discharge were found between local and distant inpatients. The significant comparisons on the age continuum and between the inpatients receiving and not receiving surgery were validated at the 99% confidence level in both linear models.

Blacks were more likely to seek local hospitalization than whites (OR = 1.24); the findings for other races/ethnicities did not differ significantly from findings for whites in logistic models (Table 1). Although the findings for Asians were significant at only the 90% confidence level (OR = 1.31), Asians were more likely to be locally hospitalized than whites. Hispanics, despite traveling 2.7% less time than whites to hospitals, spent approximately the same amount of excess travel time as whites. Other significant findings were found after changing the reference category for each factor while examining the odds of local hospitalization (Table 2) and excess travel time (Table 3). When blacks were used as the reference group in the logistic model, Hispanics were less likely to choose local hospitalization (OR = 0.83), and in the linear model they were more likely to spend 1.9% more excess travel time to hospitals.

**Table 2:** Odds of Local Hospitalization Rates for Patients Diagnosed With Congestive Heart Failure (N = 62,451), by Alternating Reference Group, Florida, United States, 2011

Factor	Reference Group					
**Race/ethnicity**	**White**	**Black**	**Hispanic**	**Asian**	**Native American**	**Other**
White	1.00	0.80 (0.76–0.85)	0.97 (0.91–1.03)	0.76 (0.57–1.02)	0.79 (0.44–1.41)	1.01 (0.83–1.21)
Black	1.24 (1.17–1.32)	1.00	1.21 (1.12–1.30)	0.95 (0.71–1.27)	0.98 (0.55–1.76)	1.25 (1.03–1.52)
Hispanic	1.03 (0.97–1.10)	0.83 (0.77–0.89)	1.00	0.78 (0.58–1.05)	0.81 (0.45–1.46)	1.04 (0.86–1.26)
Asian	1.31 (0.98–1.76)	1.06 (0.79–1.42)	1.28 (0.95–1.71)	1.00	1.04 (0.54–1.99)	1.32 (0.94–1.86)
Native American	1.29 (0.72–2.31)	1.02 (0.57–1.84)	1.23 (0.69–2.22)	0.97 (0.50–1.85)	1.00	1.28 (0.69–2.36)
Other	0.99 (0.82–1.20)	0.80 (0.66–0.97)	0.97 (0.80–1.17)	0.76 (0.54–1.07)	0.78 (0.42–1.44)	1.00
**Insurance**	**Medicare**	**Medicaid**	**Private**	**Self-pay**	**No charge**	**Others**
Medicare	1.00	0.87 (0.80–0.95)	1.30 (1.20–1.40)	0.81 (0.71–0.92)	0.79 (0.65–0.96)	1.09 (0.96–1.25)
Medicaid	1.15 (1.06–1.25)	1.00	1.49 (1.35–1.64)	0.93 (0.81–1.06)	0.91 (0.74–1.11)	1.26 (1.08–1.46)
Private	0.77 (0.71–0.83)	0.67 (0.61–0.74)	1.00	0.62 (0.54–0.71)	0.61 (0.50–0.74)	0.84 (0.73–0.98)
Self-pay	1.24 (1.09–1.40)	1.08 (0.94–1.24)	1.61 (1.41–1.84)	1.00	0.98 (0.78–1.22)	1.36 (1.14–1.62)
No charge	1.27 (1.04–1.54)	1.10 (0.90–1.35)	1.65 (1.35–2.01)	1.02 (0.82–1.28)	1.00	1.39 (1.10–1.74)
Other	0.92 (0.80–1.05)	0.80 (0.69–0.92)	1.19 (1.03–1.38)	0.74 (0.62–0.88)	0.72 (0.57–0.91)	1.00
**Location/area^a^**	**Large Metropolitan**	**Small Metropolitan**	**Micropolitan**		**Rural**	
Large metropolitan	1.00	1.06 (1.01–1.10)	1.24 (1.13–1.35)		1.98 (1.78–2.21)	
Small metropolitan	0.94 (0.90–0.99)	1.00	1.17 (1.07–1.28)		1.87 (1.68–2.09)	
Micropolitan	0.81 (0.74–0.88)	0.86 (0.78–0.94)	1.00		1.60 (1.41–1.83)	
Rural	0.50 (0.45–0.56)	0.53 (0.48–0.60)	0.62 (0.55–0.71)		1.00	
**Household income quartile**	**1st**	**2nd**	**3rd**		**4th**	
1st	1.00	1.01 (0.96–1.06)	1.09 (1.03–1.15)		1.11 (1.02–1.20)	
2nd	0.99 (0.95–1.04)	1.00	1.08 (1.03–1.14)		1.10 (1.02–1.20)	
3rd	0.92 (0.87–0.97)	0.92 (0.88–0.97)	1.00		1.02 (0.94–1.10)	
4th	0.90 (0.83–0.98)	0.91 (0.84–0.99)	0.98 (0.91–1.07)		1.00	

a A large metropolitan area was 1 million or more residents, and a small metropolitan area was fewer than 1 million residents.

**Table 3:** Standardized Coefficients of the Factors Influencing Excess Travel Time, by Patients Diagnosed With Congestive Heart Failure (N = 62,451), by Alternating Reference Group, Florida, United States, 2011

Factor	Reference Group					
**Race/ethnicity**	**White**	**Black**	**Hispanic**	**Asian**	**Native American**	**Other**
White	1	0.023	−0.002	0.049	−0.055	−0.036
Black	−0.019	1	−0.021	0.021	−0.064	−0.048
Hispanic	0.001	0.019	1	0.038	−0.039	−0.025
Asian	−0.007	−0.004	−0.007	1	−0.015	−0.012
Native American	0.004	0.006	0.004	0.008	1	0.001
Other	0.008	0.013	0.007	0.018	−0.004	1
**Insurance**	**Medicare**	**Medicaid**	**Private**	**Self−pay**	**No charge**	**Other**
Medicare	1	0.017	−0.075	0.008	0.051	−0.017
Medicaid	−0.011	1	−0.061	−0.006	0.023	−0.023
Private	0.046	0.056	1	0.051	0.078	0.035
Self-pay	−0.003	0.004	−0.034	1	0.018	−0.010
No charge	−0.013	−0.009	−0.033	−0.011	1	−0.018
Other	0.006	0.012	−0.020	0.009	0.024	1
**Location/area^a^**	**Large Metropolitan**	**Small Metropolitan**	**Micropolitan**		**Rural**	
Large metropolitan	1	−0.016	−0.183		−0.563	
Small metorpolitan	0.015	1	−0.159		−0.522	
Micropolitan	0.084	0.076	1		−0.175	
Rural	0.184	0.179	0.125		1	
**Household income quartile**	**1st**	**2nd**	**3rd**		**4th**	
1st	1	−0.020	−0.053		−0.073	
2nd	0.019	1	−0.031		−0.050	
3rd	0.047	0.029	1		−0.018	
4th	0.039	0.028	0.011		1	

a A large metropolitan area was 1 million or more residents, and a small metropolitan area was fewer than 1 million residents.

Variations in patients’ travel patterns were found by patient insurance coverage and by source of health insurance payment. The odds of local hospitalization for Medicaid, self-pay, and no-charge patients were all higher than those for Medicare patients, and privately insured patients were more likely to travel outside the HSAs (Table 1); the number of no-charge patients was only 1.4% of Medicare patients. Alternating the reference group confirmed that privately insured patients were most likely to travel outside the HSAs than any other payer group, followed by Medicare patients (Table 2). In addition to confirming that privately insured patients spent the most excess travel time, the linear model indicated that no-charge patients spent the least amount of excess travel time to hospitals compared with Medicaid and self-pay patients (Table 3), a difference that was indistinguishable in logistic models.

Patients living in large metropolitan areas were most likely to seek hospitalization within their HSAs and also spent the shortest excess travel time to hospitals (Table 1). Rural patients spent 22.4% more travel time and 18.4% more excess travel time to hospitals than large-metropolitan patients (Table 1) and 17.9% and 12.5% more excess time to hospitals than small-metropolitan or micropolitan patients, respectively (Table 3).

The differences in travel patterns of patients living in the postal zones with different median household incomes were similarly apparent. As median household income in a postal zone increased, patients were more likely to travel outside their HSA for hospitalization and spend more excess travel time to hospitals. Despite a general tendency toward distant hospitalization as median household income increased, significant differences were not found between the 2 wealthier groups and the 2 poorer groups in logistic models (Table 2). Nevertheless, linear models showed that patients in the 4th quartile of median household income spent 1.1% more excess travel time than the patients in the 3rd quartile, and 2nd-quartile patients spent 1.9% more excess travel time to hospitals than 1st-quartile patients (Table 3).

In logistic models and linear regression models controlling for all factors, 16 comorbidities were significantly associated with travel patterns. Most comorbidities were more likely to be diagnosed in local patients, whereas 5 specific comorbidities were more likely to be diagnosed in distant patients (Table 1).

## Discussion

We examined influential demographic, geographic, and socioeconomic factors associated with disparities in the travel patterns of CHF patients. To overcome the biases of a onetime decision made by patients, discharge-level data instead of patient-level data were used. More than 20% of the hospitalizations occurred outside of patients’ HSAs in 2011, and significant disparities in travel patterns among different groups of inpatients were found, which were confirmed by the significant relationships of those influential factors to excess travel time.

Choosing a hospital can be a difficult decision, especially in large cities where many choices are available to most residents that are within an acceptable distance or travel time. Unlike many traditional studies, we did not focus on which groups of patients lacked access to health care resources. Instead, assuming that most patients had access to local hospital resources and that HSAs were delineated on the basis of acceptable distances for most patients, we defined local hospitalization as a preference of going to the closest hospital rather than being forced to go. Given that patients’ accurate addresses are often not available because of privacy protection, examining the tendency to choose close hospitals is more meaningful than scrutinizing whether patients go to the physically closest hospital (16).

In addition to using a dichotomous variable and traditional continuous travel time, the concept of local hospitalization was also used as a dependent variable in linear regression models (excess travel time) to supplement the results from logistic models. Excess time removed the effects of varying closest hospitals and, compared with travel time, better reflected the tendency of the patients to travel. For example, although spending as much travel time as blacks, Hispanics spent 1.9% more excess travel time, which implies that Hispanics may live closer to hospitals but bypass their closer choices to a larger extent than blacks do.

According to the results from logistic models, the subgroups most (least) likely to stay within the HSAs were blacks (whites); Medicaid, self-pay, and no-charge patients (privately insured patients); large metropolitan patients (rural patients); and patients in the 1st and 2nd income quartiles (3rd- and 4th-quartile patients). The juxtaposed subgroups were statistically indistinguishable in logistic models, but they were significantly different in the linear models, producing 3 new findings: 1) no-charge patients spent less time on travel than did Medicaid and self-pay patients, 2) patients in the 1st income quartile patients spent less time than those in the 2nd quartile, and 3) patients in the 3rd income quartile patients spent less time than the those in the 4th quartile. Therefore, the 2 approaches complement each another while providing consistent evidence, and the combination of logistic and linear regression, in conjunction with using dummy variables converted from categorical variables, could be applied to future research.

Our results indicate that patients’ SES plays a prominent role in their travel behavior, possibly by providing them more choices or a stronger tendency to travel. Blacks and Hispanics normally have an apparent disadvantage in SES relative to other races/ethnicities, and they are more likely to live in the inner city and rely on hospitals around them, which may explain their shorter excess travel time. Furthermore, because Medicare and Medicaid patients do not have the burden of payment, they often have more choices. However, being eligible for Medicaid is an indicator of low SES, which accounts for these patients being less likely to travel farther than their Medicare counterparts. No charge for hospitalization also indicates low SES, so SES may underlie both geographic and payer factors. Another possible explanation is the interplay between the insured and uninsured. No-charge patients may prefer to go to local hospitals they are more familiar with and save the costs of traveling, and self-pay patients may prefer local hospitals for a discount received by paying cash.

A major limitation of this study is that patients’ residential location can only be determined by zip code where they lived at the time of discharge, because of data confidentiality laws. We assumed that the zip codes inpatients claimed as residential zip codes are those of their own permanent addresses or that influenced their choices of travel distance or time (such as those of extended family). The small sample size in some subpopulations may affect the conclusions, especially the comparisons with other subpopulations under the same category. For example, the higher tendency of Asians to be locally hospitalized might be discounted by a small sample size (ie, only 0.74% of whites). Also, our focus was the tendency of patients to seek hospitalization with travel time, insurance, and SES considered, regardless of whether those inpatient visits were transferred to current hospitals. Therefore, we assume that those transferred to current hospitals do not significantly affect our conclusions.

Other limitations are lack of consideration for hospital quality and capacity and insurance network within the HSAs. For example, renowned hospitals and those with sufficient number of beds within the HSAs influence patients to choose local hospitalization. Some types of insurance, such as Medicare Advantage plans, dictate a network in which patients may receive services, making some hospitals unavailable to nearby populations. Nevertheless, we emphasized the concept of local hospitalization instead of the closest hospital, which reduces this bias. Compared with metropolitan areas, rural areas may be disproportionately affected because of fewer choices within the HSAs. The delineation of HSAs is also subject to the modifiable areal unit problem (MAUP) (23), which should be further explored in future studies. The results related to Native Americans and Asians should be interpreted with caution because of small numbers and percentages over the entire population. Patients’ travel patterns may vary across diagnoses and diagnostic procedures (4,24). Even if the severity of illness is controlled, the pattern may vary geographically. Consequently, results of this study should be interpreted with caution when applied to patients diagnosed with other symptoms or those in other states or regions.

Study results imply that changes or disruptions in local hospital supply, such as hospital closures or changes in hospital resource allocation and transportation infrastructures, could differentially affect different population groups, which may require policy makers to pay extra attention to the disadvantaged populations who are less likely to travel. Furthermore, the HSA has re-emerged as an important unit of analysis, especially after the 2013 report by the Institute of Medicine (25). Future research should explore the value of HSAs in maximizing the efficiency of health care systems while reducing costs.

## References

[1] Pfuntner A , Wier LM , Stocks C . Most frequent conditions in US hospitals, 2010. In: Statistical brief no. 148. Rockville (MD): Agency for Healthcare Research and Quality; 2013. 23534077

[2] Wang F . Measurement, optimization, and impact of health care accessibility: a methodological review. Ann Assoc Am Geogr 2012;102(5):1104–12. 10.1080/00045608.2012.657146 23335813 PMC3547595

[3] Jia P , Xierali I , Wang F . Evaluating and re-demarcating the hospital service areas in Florida. Appl Geogr 2014;60:248–53. 10.1016/j.apgeog.2014.10.008

[4] Hogan C . Patterns of travel for rural individuals hospitalized in New York State: relationships between distance, destination, and case mix. J Rural Health 1988;4(2):29–41. 10.1111/j.1748-0361.1988.tb00310.x 10312687

[5] Wilbush J . The local hospital: functions, advantages and difficulties. Can Fam Physician 1974;20(7):54–6. 20469088 PMC2274218

[6] Moist LM , Bragg-Gresham JL , Pisoni RL , Saran R , Akiba T , Jacobson SH , Travel time to dialysis as a predictor of health-related quality of life, adherence, and mortality: the Dialysis Outcomes and Practice Patterns Study (DOPPS). Am J Kidney Dis 2008;51(4):641–50. 10.1053/j.ajkd.2007.12.021 18371540

[7] Nicholl J , West J , Goodacre S , Turner J . The relationship between distance to hospital and patient mortality in emergencies: an observational study. Emerg Med J 2007;24(9):665–8. 10.1136/emj.2007.047654 17711952 PMC2464671

[8] Jones AP , Haynes R , Sauerzapf V , Crawford SM , Zhao H , Forman D . Travel time to hospital and treatment for breast, colon, rectum, lung, ovary and prostate cancer. Eur J Cancer 2008;44(7):992–9. 10.1016/j.ejca.2008.02.001 18375117

[9] Gabrysch S , Campbell OM . Still too far to walk: literature review of the determinants of delivery service use. BMC Pregnancy Childbirth 2009;9(1):34. 10.1186/1471-2393-9-34 19671156 PMC2744662

[10] Masters SH , Burstein R , Amofah G , Abaogye P , Kumar S , Hanlon M . Travel time to maternity care and its effect on utilization in rural Ghana: a multilevel analysis. Soc Sci Med 2013;93:147–54. 10.1016/j.socscimed.2013.06.012 23906132

[11] Basu J , Friedman B . A re-examination of distance as a proxy for severity of illness and the implications for differences in utilization by race/ethnicity. Health Econ 2007;16(7):687–701. 10.1002/hec.1192 17191272

[12] Basu J . Severity of illness, race, and choice of local versus distant hospitals among the elderly. J Health Care Poor Underserved 2005;16(2):391–405. 10.1353/hpu.2005.0023 15937400

[13] Basu J , Cooper J . Out-of-area travel from rural and urban counties: a study of ambulatory care sensitive hospitalizations for New York State residents. J Rural Health 2000;16(2):129–38. 10.1111/j.1748-0361.2000.tb00446.x 10981364

[14] Biello KB , Rawlings J , Carroll-Scott A , Browne R , Ickovics JR . Racial disparities in age at preventable hospitalization among U.S. Adults. Am J Prev Med 2010;38(1):54–60. 10.1016/j.amepre.2009.08.027 20117557 PMC2828489

[15] O’Hare AM , Johansen KL , Rodriguez RA . Dialysis and kidney transplantation among patients living in rural areas of the United States. Kidney Int 2006;69(2):343–9. 10.1038/sj.ki.5000044 16408125

[16] Radcliff TA , Brasure M , Moscovice IS , Stensland JT . Understanding rural hospital bypass behavior. J Rural Health 2003;19(3):252–9. 10.1111/j.1748-0361.2003.tb00571.x 12839133

[17] Delmelle EM , Cassell CH , Dony C , Radcliff E , Tanner JP , Siffel C , Modeling travel impedance to medical care for children with birth defects using Geographic Information Systems. Clin Molec Teratology 2013;97(10):673–84. 10.1002/bdra.23168 PMC4507419 23996978

[18] Basu J , Mobley LR . Illness severity and propensity to travel along the urban-rural continuum. Health Place 2007;13(2):381–99. 10.1016/j.healthplace.2006.03.002 16697689

[19] Lembcke PA . Measuring the quality of medical care through vital statistics based on hospital service areas; I. Comparative study of appendectomy rates. Am J Public Health Nations Health 1952;42(3):276–86. 10.2105/AJPH.42.3.276 14903245 PMC1526005

[20] Klauss G , Staub L , Widmer M , Busato A . Hospital service areas — a new tool for health care planning in Switzerland. BMC Health Serv Res 2005;5(1):33. 10.1186/1472-6963-5-33 15882463 PMC1131901

[21] Healthcare Cost and Utilization Project. Overview of the State Inpatient Databaes. Agency for Healthcare Research and Quality; 2011. http://www.hcup-us.ahrq.gov/sidoverview.jsp. Accessed August 11, 2015.

[22] Luo W , Wang F . Measures of spatial accessibility to health care in a GIS environment: synthesis and a case study in the Chicago region. Environ Plan B 2003;30(6):865–84. 10.1068/b29120 PMC8238135 34188345

[23] Fotheringham AS , Wong DW . The modifiable areal unit problem in multivariate statistical analysis. Environ Plan A 1991;23(7):1025–44. 10.1068/a231025

[24] Mayer JD . The distance behavior of hospital patients: a disaggregated analysis. Soc Sci Med 1983;17(12):819–27. 10.1016/0277-9536(83)90032-1 6879240

[25] Newhouse JP , Garber AM , Graham RP , McCoy MA , Mancher M , Kibria A . Variation in health care spending: target decision making, not geography. Washington (DC): Institute of Medicine, National Academies Press; 2013. 24851301

